# Noninvasive Fetal Trisomy (NIFTY) test: an advanced noninvasive prenatal diagnosis methodology for fetal autosomal and sex chromosomal aneuploidies

**DOI:** 10.1186/1755-8794-5-57

**Published:** 2012-12-01

**Authors:** Fuman Jiang, Jinghui Ren, Fang Chen, Yuqiu Zhou, Jiansheng Xie, Shan Dan, Yue Su, Jianhong Xie, Baomin Yin, Wen Su, Huakun Zhang, Wei Wang, Xianghua Chai, Linhua Lin, Hui Guo, Qiyun Li, Peipei Li, Yuying Yuan, Xiaoyu Pan, Yihan Li, Lifu Liu, Huifei Chen, Zhaoling Xuan, Shengpei Chen, Chunlei Zhang, Hongyun Zhang, Zhongming Tian, Zhengyu Zhang, Hui Jiang, Lijian Zhao, Weimou Zheng, Songgang Li, Yingrui Li, Jun Wang, Jian Wang, Xiuqing Zhang

**Affiliations:** 1 BGI- Shenzhen, Shenzhen, China; 2 The Center of Prenatal Diagnosis, Shenzhen People’s Hospital, 2nd Clinical Medical College of Jinan University, Shenzhen, Guangdong, China; 3 Zhuhai Institute of Medical Genetics, Zhuhai Municipal Maternal and Child Healthcare Hospital, Zhuhai, China; 4 Central for Prenatal Diagnosis, Shenzhen Maternity and Child Healthcare Hospital, Affiliated Southern Medical University, Shenzhen, China; 5 Department of Perinatology, Beijing Obstetrics and Gynecology Hospital-Capital University of Medical Sciences, Beijing, China

**Keywords:** Noninvasive Fetal Trisomy (NIFTY) test, Massively parallel sequencing, Autosomal aneuploidies, Sex chromosomal aneuploidies

## Abstract

**Background:**

Conventional prenatal screening tests, such as maternal serum tests and ultrasound scan, have limited resolution and accuracy.

**Methods:**

We developed an advanced noninvasive prenatal diagnosis method based on massively parallel sequencing. The Noninvasive Fetal Trisomy (NIFTY) test, combines an optimized Student’s t-test with a locally weighted polynomial regression and binary hypotheses. We applied the NIFTY test to 903 pregnancies and compared the diagnostic results with those of full karyotyping.

**Results:**

16 of 16 trisomy 21, 12 of 12 trisomy 18, two of two trisomy 13, three of four 45, X, one of one XYY and two of two XXY abnormalities were correctly identified. But one false positive case of trisomy 18 and one false negative case of 45, X were observed. The test performed with 100% sensitivity and 99.9% specificity for autosomal aneuploidies and 85.7% sensitivity and 99.9% specificity for sex chromosomal aneuploidies. Compared with three previously reported z-score approaches with/without GC-bias removal and with internal control, the NIFTY test was more accurate and robust for the detection of both autosomal and sex chromosomal aneuploidies in fetuses.

**Conclusion:**

Our study demonstrates a powerful and reliable methodology for noninvasive prenatal diagnosis.

## Background

Down syndrome (Trisomy 21), Edward syndrome (Trisomy 18) and Patau syndrome (Trisomy 13) are the most clinically significant autosomal aneuploidies, and the incidence of autosomal abnormalities can be as high as one in 160 live births [1]. Turner’s syndrome (45, X), Klinefelter’s syndrome (47, XXY) and XYY syndrome are common sex chromosomal aneuploidies that are associated with reproductive loss, infertility and language development delays, among others [2-4]. Sex chromosomal aneuploidies occur in one out of 500 male births and one out of 850 female births [5-8].

Conventional prenatal diagnostic methods for detecting aneuploidies, such as karyotyping, FISH and QF-PCR, which rely on invasive procedures, bear potential risks for miscarriage [9,10]. Noninvasive screening for fetal aneuploidies using maternal serum markers and ultrasound scans entails less risk, but offers limited sensitivity and specificity [11,12].

When Lo et al. first reported cell-free fetal DNA (cff-DNA) in 1997 [13], and they highlighted its potential clinical utility as a biomarker because it can be detected from as early as four gestational weeks. Cell-free fetal DNA clears rapidly from the maternal circulation after delivery [14-16]. However, the fraction of fetal DNA in the maternal plasma varies from 5% to 10%, which makes it difficult to detect genetic variation in the fetus [17,18]. Conventional molecular techniques, such as allele-specific polymerase chain reaction (PCR) or quantitative real-time PCR, which aim to detect fetal chromosomal disorders, focus only on specific populations [19-21], such as fetuses with heterozygous alleles. The recent rapid development of massively parallel sequencing (MPS) technology now makes it possible to noninvasively detect fetal aneuploidies in a clinical setting [22-24]. Several recent studies demonstrated that fetal aneuploidies could be detected and quantified via high-throughput whole-genome sequencing of maternal plasma cell-free DNA combined with a standard z-score test. Prior studies by Chiu et al. and Ehrich et al. suggest that an MPS-based approach is reliable at detecting trisomy 21 [25,26].

In principle, an MPS-based approach that resolves whole genome information should be applicable for detecting aneuploidies in all of the chromosomes. Chen et al. showed, however, that such test was less successful for detecting trisomy 18 and trisomy 13 compared with trisomy 21. The mixed results may be related to the GC-bias caused by the sample preparation or sequencing procedures [27]. Quake et al. developed a method to remove the effect of GC-bias, and thus significantly improve the sensitivity of the MPS-based approach for detecting trisomy 18 and trisomy 13 [28]. Another recent study reported the possibility of detecting sex chromosomal aneuploidies using an internal chromosome control approach [29].

In this study, we developed an advanced GC-correlation methodology for an MPS-based, noninvasive fetal trisomy (NIFTY) test. Our technique has higher sensitivity and specificity than all previously reported z-score approaches for the detection of autosomal and sex chromosomal aneuploidy.

## Results

### Study participants and data production

**Figure 1:**
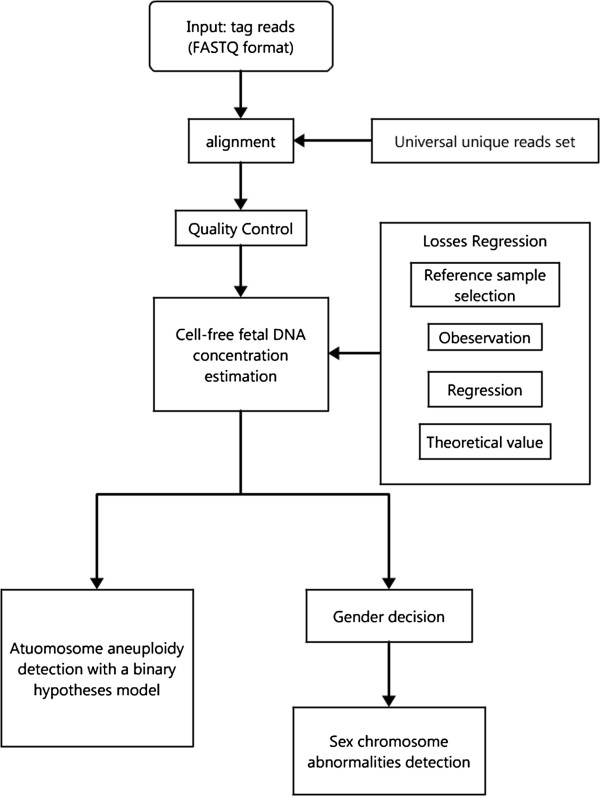
**The flowchart of the whole bioinformatics pipeline.** A comprehensive bioinformatics pipeline, including effective short read alignment, quality control, data correction, cell-free fetal DNA concentration estimation, and aneuploidy detection.

We enrolled 903 pregnant women with ages ranging from 20 to 45 years. The gestational ages varied from 10 to 34 weeks, covering the first to the third trimesters. Based on the results of full karyotyping using amniotic fluid, 866 of the fetuses were euploid and 37 were aneuploid. The cases of aneuploidy included two cases of trisomy 13, 12 cases of trisomy 18, 16 cases of trisomy 21, four cases of 45,X (three typical cases of 45,X and one mosaic case of 45, X ([27]/46, XX [23]), two cases of XXY and one case of XYY. We obtained 2–4 million reads for each sample. After alignment and filtering, the average data volume for aneuploidy detection was 1.7 million uniquely aligned reads. We constructed a comprehensive bioinformatics pipeline to scan for fetal chromosomal aneuploidies. The pipeline comprised short reads alignment, GC content correction, fetal DNA concentration estimation, t-test of a binary hypothesis, and fetal gender classification (Figure 1).

### The relationship between GC content and sequencing bias

To investigate the relationship between GC content and sequence bias, we selected 300 control pregnancies with normal karyotypes and plotted the relative reads coverage for each chromosome against the corresponding GC content (Additional file 1: Figure S1). The relative reads coverage of the different chromosomes was strongly related to the inherent chromosomal GC content, and the correlation between the two factors varied among the chromosomes. We observed a significant positive correlation between the reads coverage and GC content for chromosomes with an average GC content greater than 41%, whereas we observed, a significant negative correlation between the reads coverage and GC content for chromosomes with an average GC content less than 41%. Among the chromosomes with average GC content close to 41% the reads coverage was not correlated with GC content (Additional file 2: Figure S2). To further investigate the effect of GC content on reads coverage, we examined the hidden relationship between chromosome structure and inherent GC content. We classified all of the unique 35-mers in the genome into 36 levels based on the numbers of guanine (G) and cytosine (C) bases, ranging from 0 to 35. We used the 35-mer counts to cluster the chromosomes according to their GC levels within a matrix (Additional file 3: Figure S3). Chromosomes 19 and 22 clustered together because of their higher inherent GC contents, while chromosome 4 and 13 clustered together for their lower inherent GC contents. The differences in the inherent GC content of the chromosomes combined with the sequencer-related GC-bias explained the significant correlation between reads coverage and corresponding GC content. For example, chromosome 13 has a relatively low GC content, the PCR and sequencing process enriched chromosomes with higher GC content, leading to relatively low reads coverage for chromosome 13 and thus a negative correlation between the reads coverage and GC content among the chromosomes (Additional file 1: Figure S1).

### The relationship between cell-free fetal DNA concentration and gestational week

Previous work demonstrated that the cff-DNA concentration was the lynchpin in fetus aneuploidy detection [16]. To assess the best strategy for clinical applications, we examined the relationship between cell-free fetal DNA concentration and the gestational week. We examined the 443 plasma samples with male fetal to appraise the approximate probability relationship. Using Losses regression, we found that the amount of cff-DNA increased significantly with the gestational week (Additional file 4: Figure S4). The correlation coefficient of the linear regression was only 0.1246, however, indicating that there might be a more complex mechanism driving the cff-DNA concentrations.

### Quantitative description of data volatility and tags number

The volatility of the relative reads coverage was one of the major factors affecting the sensitivity and specificity of aneuploidy detection. To quantify the volatility of the relative reads coverage, we used the standard deviation of the difference between the observed and fitted relative reads coverage. We found that for each chromosome the standard deviation was stable when the numbers of samples was larger than 100 (Additional file 5: Figure S5). We also found that the depth of sequencing strongly influenced the accuracy of aneuploidy detection. We isolated 150 plasma samples with euploid fetuses to inspect the relationship between the tags number (unique reads) and the standard deviation of relative reads coverage. On each chromosome, the standard deviation of relative reads coverage among the 150 samples was significantly correlated with the numbers of tags (Additional file 6: Figure S6). We further estimated the effects of the gestational week and the number of tags on the power of our statistical method to detect fetal aneuploidy in chromosomes 13, 18, 21 and X (Additional file 7: Figure S7). In most cases, the detection power increased with both gestational week and the number of sequencing reads. We also found that the detection power was higher when the fetus was male; it may be due to the more accurate estimation of cff-DNA concentration.

### Robust data quality control of the GC-correlation t-test

**Figure 2:**
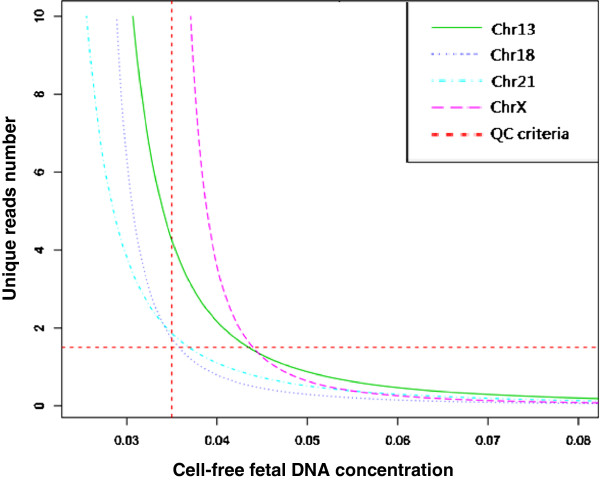
**The required number of of unique reads for high sensitivity across different cff-DNA concentrations.** For aneuploidy detection, the required number of unique reads (y-axis) increased with decreasing cff-DNA concentration (x-axis). For a 3.5% cff-DNA concentration, 1.7 million unique reads are needed to obtain high sensitivity.

Several indicators were used to judge the quality of the sequence data. We classified these indicators into two categories: direct and indirect. The indirect indicators of the accuracy of NIFTY test came from the sequencing procedure: Q20% refers to the fraction of bases within the sequenced reads with an Illumina quality score greater than 20, and the PCR duplication rate, refers to the fraction of the reads sharing the same start position and end positions on the reference genome. The direct indicators came from the data analysis procedure and included the number of unique reads (Figure 2), the genome-wide average GC content, and the consistency between the test samples and the reference controls.

### Aneuploidy detection with NIFTY test

**Figure 3:**
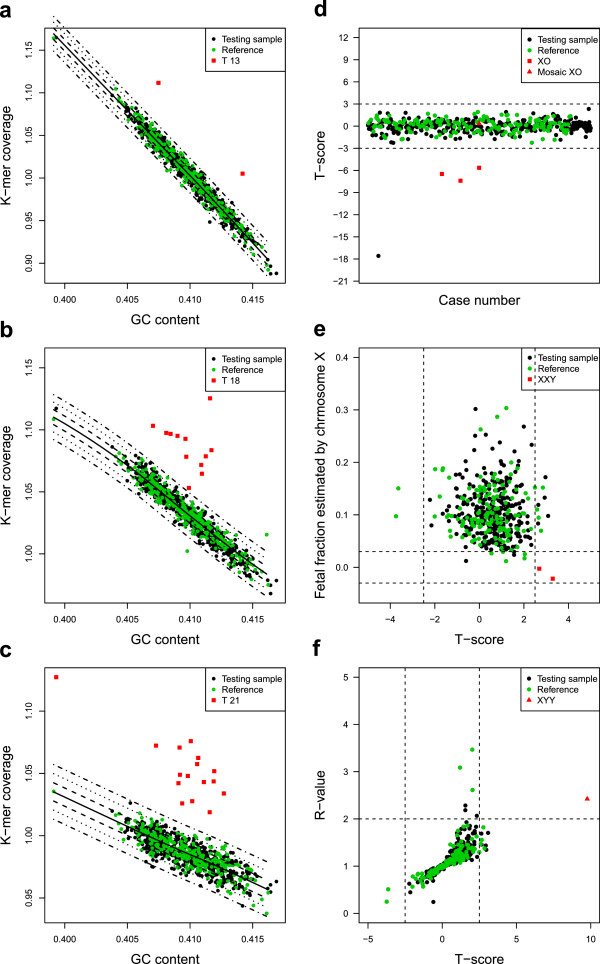
**Fetal aneuploidy detection using NIFTY test. a-c**, The k-mer coverage (y-axis) of 903 samples was plotted with corresponding GC content (x-axis) for chromosome 13,18 and 21. The solid black line is the fit between the k-mer coverage and GC content among the 300 controls. The dot-dash lines from inside to outside are the contour lines of t=1, t=2 and t=3 respectively. **d**, XO detection. The t-score of chromosome X for 452 cases with female fetuses and 4 XO cases is dotted. The t-score less than −2.5 indicates XO aneuploidy. **e**, XXY detection. The x-axis is the t-score of chromosome X for samples carrying male fetuses. The y-axis is the fetal fraction estimated by chromosome X. Red square points indicated XXY cases that have a t-score larger than 2.5 and the cff-DNA concentration estimated by chromosome X nearly equal to zero. **f**, XYY detection. The x-axis is t-score for chromosome X among samples carrying male fetuses. The y-axis is the R-value, i.e. the ratio of the fetal DNA fraction estimated by chromosome Y to that estimated by chromosome X. Red triangle points indicate XYY cases with t-score greater than 2.5 and R-value greater than 2. The case types are color coded (black: testing samples; green: reference samples; red: aneuploidy samples).

The NIFTY test performed with 100% sensitivity and specificity for the detection of trisomy 13 (two out of two) (Figure 3a) and trisomy 21 (16 out of 16) (Figure 3c). For trisomy 18, the NIFTY test detected 12 of 12 cases and identified 890 of 891 healthy controls (Figure 3b), indicating 100% sensitivity and 99.7% specificity, corresponding to zero false negative results and a false positive rate of 0.3%. The false positive occurred in a sample from gestational week 21. Our GC-correlation t-test approach correctly detected sex chromosomal abnormalities. For Turner’s syndrome, the NIFTY test identified three out of four XO cases but failed to detect the mosic 45, X case which was in gestational week of 25 and had a normal karyotype in 46% of the cells sampled. Thus, the sensitivity and specificity of our approach for the detection of Turner’s syndrome were 75% and 99.9%, respectively; in other words, the false negative rate was 25% and false positive rate was 0.1% for 45, X detection using the NIFTY test. The test performed with 100% sensitivity and specificity for the detection of XXY (two out of two) or XYY (one out of one) (Figure 3e and f).

The NIFTY test correctly identified the sex of approximately 99.9% of the 896 fetuses, 443 male and 452 female, which did not have sex chromosomal aneuploidies. The NIFTY test was inconclusive for one fetus that was determined to be 46, XX by karyotyping.

### Comparison between different aneuploidy detection approaches

To evaluate the performance of the NIFTY test in the detection of fetal aneuploidy, we compared it with the performance of three other previously reported approaches to analyse our 903 cases, with full karyotyping of the same 300 euploid cases [23,27,29]. Chiu et al. used the standard z-score approach without any GC-bias removal to detect Down syndrome [23]. Chen et al. developed a z-score approach with a different GC-bias removal strategy [27], which we named the “GC-correct z-score approach.” Lau et al. previously demonstrated a internal chromosome control based z-score approach [29].

**Figure 4:**
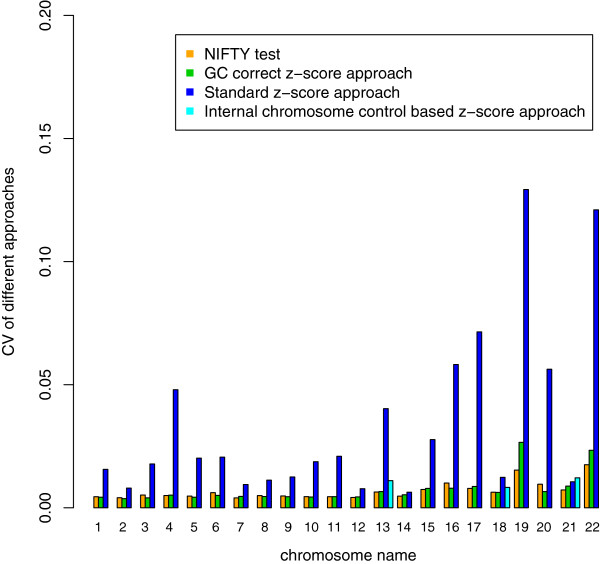
**The Performance of three methods: coefficient of variation (CV).** We calculated the CVs (y-axis) among the different chromosomes (x-axis) for the 903 samples with karyotyping. The different methods are the color-coded (Orange: NIFTY test; Green: GC correct z-score approach; Dark Blue: Standard z-score approach; Light Blue: Internal chromosome control based z-score approach). In the clinically interesting chromosomes (13, 18, 21), our approach obtained the lowest CVs, indicating a higher sensitivity.

We used the coefficient of variation (CV) to evaluate the performances of these four approaches (Figure 4). Additionally, we found that the CV for the standard z-score approach was larger than that for other approaches among clinically relevant chromosomes (13, 18 and 21). Thus, the standard z-score approach has a low sensitivity for the detection of trisomies 13 and 18 (Table 1). The performance of the GC-correct z-score approaches and our NIFTY test were close, both demonstrated over 99% sensitivity and specificity for the detection of trisomy 13, 18 and 21 (Table 1). It was difficult to precisely detect sex chromosomal aneuploidy using the GC-correct z-score approach due to fetal gender confusion. The internal chromosome control approach displayed larger CV values for chromosomies 13, 18, and 21 and had a higher risk of false negatives related to XXY and XYY detection. In contrast, the NIFTY test had increased accuracy in the detection of sex chromosomal aneuploidies, such as XO, XXY and XYY (Table 1).

**Table 1:** The performance of four approaches for detection of fetal aneuploidy

**Test**		**Standard z-score approach**		**GC correct z-score approach**		**Internal chromosome control based z-score approach**		**NIFTY test**	
**(Number of cases)**		**Sensitivity**	**Specificity**	**Sensitivity**	**Specificity**	**Sensitivity**	**Specificity**	**Sensitivity**	**Specificity**
Autosome	T13 (2)	50%(1/2)	99.9%(900/901)	100%(2/2)	100%(901/901)	100%(2/2)	99.7%(898/901)	100%(2/2)	100%(901/901)
	T18 (12)	91.7%(11/12)	100%(891/891)	100%(12/12)	99.9%(890/891)	100%(12/12)	100%(891/891)	100%(12/12)	99.9%(890/891)
	T21 (16)	93.7%(15/16)	100%(887/887)	100%(16/16)	100%(887/887)	100%(16/16)	100%(887/887)	100%(16/16)	100%(887/887)
Sex chromosome*	45, X (3 45, X, 1 45,X/46,XX)	Not available	Not available	Not available	Not available	75%(3/4)	99.8%(897/899)	75%(3/4)	99.9%(898/899)
	XYY (1)	Not available	Not available	Not available	Not available	0%(0/1)	100%(902/902)	100%(1/1)	100%(902/902
	XXY (2)	Not available	Not available	Not available	Not available	0% (0/2)	100%(901/901)	100%(2/2)	100%(901/901)
Binary hypothesis		Not available		Not available		Available		Available	

* We could not perform sex chromosome aneuploidy detection for one case because of the failure in gender classification.

## Discussion

The cost of high throughput sequencing decreased dramatically over the past few years, thus increasing its utility for clinical practices [30,31]. In this study, we demonstrated the NIFTY test is a novel MPS-based method for noninvasively detecting fetal aneuploidies. the NIFTY test proved to be a reliable and timely method for detecting both autosomal and sex chromosomal aneuploidies, especially trisomy 21, trisomy 18, trisomy 13, XO, XXY and XYY.

GC-bias is a common issue for applications using the current massively parallel sequencing platforms, and it can be introduced either by sample preparation or the sequencing procedure [32,33]. In this study, we observed that sequencers appear to have a preferred GC ranges, and the differences in GC composition among different chromosomes can act as an intrinsic factor influencing the degree of data fluctuation. We employed GC-correlation to significantly reduce the effect of GC-bias. Using the same reagents for library construction and sequencing, and improving the image analysis software may be useful in further minimizing the GC-bias.

The small fetal DNA fraction in the maternal blood was the main limitation on sequencing dependent noninvasive prenatal diagnosis. The required number of unique reads increases exponentially when the concentration of cff-DNA falls to less than 3.5%, which is consistent with the results of a previous study [27]. An advantage of our approach is that the quality-control procedure uses the estimation of cff-DNA concentration as a key index. Thus, the quality-control procedure, improves the accuracy of fetal aneuploidy detection. Another improvement of our methodology over previous methods is the change from sole reliance on a binary hypothesis to a more comprehensive statistical model. Our statistical model strengthens the theoretical sensitivity of the test; the NIFTY test performed with 100% sensitivity and 99.9% specificity for detecting autosomal aneuploidies and 85.7% sensitivity and 99.9% specificity for detecting sex chromosomal aneuploidies.

Using the NIFTY test we treated each chromosome as a whole, which allowed us to focus on detecting the aneuploidies. In principle, we could detect other chromosomal abnormalities resulting in serious clinical consequences, such as microdeletions and microduplications, by slicing the chromosomes into smaller fragments and increasing the number of sequencing tags. It is also possible to detect mutations, such as those underlying Mendelian diseases, in genes or regions of interest through target region capture and high depth sequencing [34].

Although we achieved high detection accuracy in a cohort of 903, the sample size in this study was a limiting factor because the incidence of aneuploidies in the general population is low. To precisely estimate sensitivity and specificity of our procedure, large-scale, multi-center clinical trials will be required in the future. Additionally, the conventional approaches for cff-DNA concentration estimation are mostly locus-specific and only applicable to limited population [20,35]. Our approach was also less accurate in assessing the cff- DNA concentration for female fetuses. Further studies should focus on developing an unbiased method to precisely estimate the fraction of cff-DNA in the maternal plasma.

## Conclusions

In this study we demonstrate a robust and accurate methodology to detect fetal aneuploidies using MPS. This is the first study to systematically identify sex chromosomal aneuploidies with maternal plasma DNA sequencing. We hope the use of this method in clinical practices will contribute to reducing the number of birth defects.

## Methods

### Sample recruitment

From June 2009 to August 2010, we recruited a total of 903 participants prospectively from the Shenzhen People’s Hospital, the Zhuhai Municipal Maternal and Child Healthcare Hospital and the Shenzhen Maternal and Child Care Center. We recruited another 19 euploid adult males for the estimation of fetal DNA fraction. Institutional Review Board approval was obtained at each site, and all participants gave informed written consent. We obtained the full karyotyping results for all samples from regular clinical tests. We randomly selected 300 euploid samples among the karyotyping results to use as the reference controls.

### Maternal plasma DNA sequencing

We collected five ml peripheral venous blood from 903 pregnant women in EDTA tubes. The tubes were centrifuged at 1,600 × g for 10 min within four hours of collection. Plasma was transferred to microcentrifuge tubes and centrifuged at 16,000 × g for 10 min to remove residual cells. Cell-free plasma was stored at −80°C until DNA extraction. Each plasma sample was frozen and thawed only once.

For massively parallel genomic sequencing, DNA fragments from 600 ul of maternal plasma were used for library construction according to a modified protocol from Illumina. End-repairing of maternal plasma DNA fragments was performed using T4 DNA polymerase, Klenow polymerase, and T4 polynucleotide kinase. Afterwards, A-base tailing adapters were ligated to the DNA fragments. Standard multiplex primers were introduced by 17-cycle PCR. The libraries were analysed for size distribution by Agilent Bioanalyzer and quantified using real-time PCR. Thirty-six-cycle single-end multiplex sequencing and 50-cycle single-end multiplex sequencing were used for the Illumina GAIIx and Illumina HiSeq 2000 platform, respectively.

### High effective alignment with universal unique reads set

Computationally, we incised the human reference genome (HG 18, NCBI build 36) into k-mers (k refers to the length of the sequencing reads) and then aligned the k-mers back to the reference genome. All of the k-mers that could be uniquely mapped to a single position on the reference genome, the unique mapping reads, were named as the universal unique reads set. We selected the sequencing reads that could be mapped with 0-mismatch to the universal unique reads set (i.e. the tag) for our analysis.

### K-mer coverage and GC-correlation

We computed the k-mer coverage for each chromosome and every sample, as $C_{i,j}=\frac{n_{i,j}}{N_{i}}$ where is the ID of control samples; j is the chromosome ID; *ni,j* is the number of unique reads mapped onto chromosome j from sample i and *Ni,j* was the total number of unique reads for chromosome j. Because of the differences among the samples, we normalized the data and computed the relative k-mer coverage for each sample as $r_{i,j}=\frac{C_{i,j}}{C_{i}}$, where ${\overset{–}{C}}_{i}=\frac{1}{22}\sum_{j=1}^{22}C_{i,j}$ was the average *k*-mer coverage of the 22 autosomes in the i-th sample.

Given the unclear mechanism of GC-bias, we performed a Losses regression to fit the relative k-mer coverage to the corresponding GC content. We denoted the fitted relative *k*-mer coverage as *cr*^′^_*i*,*j*_ = *f*_*j*_(*GC*_*i*,*j*_). The fitted value, which we used as the theoretical value, was vital to our statistical model for cff-DNA concentration estimation and aneuploidy detection.

Because we using a male/female data set, we had different fitted values for the analysis of sex chromosomes. We calculated the fitted relative *k*-mer values for the sex chromosome analysis as follows:

*cr*^′^_*i*,*j*,*m*_ = *f*_*j*,*m*_(*GC*_*i*,*j*_) · (*j* = *X*, *Y*), for the fitted relative *k*-mer coverage from a regression of an adult male data set; and

*cr*^′^_*i*,*j*,*f*_ = *f*_*j*,*f*_(*GC*_*i*,*j*_) · (*j* = *X*, *Y*), for the fitted relative *k*-mer coverage from a regression of a fetal-female data set.

### Cff-DNA concentration estimation

Using the gender difference to compute the relative *k*-mer coverage of the sex chromosome, we estimated the cff-DNA concentrations, which denote as ε. Subscripts corresponding to chromosome IDs indicate concentrations estimated from different chromosomes:

$\varepsilon_{i,Y}=\frac{cr_{i,Y}-c{r^{\prime }}_{i,Y,f}}{c{r^{\prime }}_{i,Y,m}-c{r^{\prime }}_{i,Y,f}}$, is the estimation using the data for chromosome Y; and

$\varepsilon_{i,X}=\frac{cr_{i,X}-c{r^{\prime }}_{i,Y,f}}{c{r^{\prime }}_{i,Y,m}-c{r^{\prime }}_{i,Y,f}}$, is the estimation using data for chromosome X.

### Autosomal aneuploidy detection with binary hypothesis

We developed a binary hypothesis strategy to achieve a higher sensitivity and specificity. We performed two Student’s t-test based on null/alternative hypotheses, and we subsequently calculated the relative logarithmic likelihood odds ratio. The null and alterative hypothesizes are shown below.

For the first test:

H0 (null hypothesis): the fetal chromosome was euploid.

H1 (alterative hypothesis): the fetal chromosome was trisomic.

The first t-value, $t_{i,j,\text{first}}=\frac{cr_{i,j}-c{r^{\prime }}_{i,j}}{sd_{j}}$.

For the second test:

H0 (null hypothesis): the test fetal chromosome was trisomic.

H1 (alterative hypothesis): the test fetal chromosome was euploid.

The second t-value, $t_{i,j,\text{second}}=\frac{cr_{i,j}-c{r^{\prime }}_{i,j}\left(1+\varepsilon_{i}/2\right)}{sd_{j}}$.

The logarithmic likelihood odds ratio between our binary hypotheses was defined as

(1) $$L_{i,j}=\text{log}\left(\frac{p\left(t_{i,j,\text{fiirst}},\text{DOF}|H_{0}\right)}{p\left(t_{i,j,\sec ond},\text{DOF}|H_{1}\right)}\right)$$

where DOF = the degree of freedom., We used, │*t*_*i,j,firsti,j,first*_│> 3 and │*t*_*i,j,secondi,j,second*_│< 3 as warning criteria. From the logarithmic likelihood odds ratio, we could make a confident judgment of autosomal aneuploidy if *L*_*i,j*_ > 1.

### Fetal gender classification and sex chromosomal aneuploidy detection

We developed a double standard strategy with an experimental threshold and logistic regression to detect the fetal gender. The k-mer coverage on chromosome Y was an ideal choice for distinguishing genders. Based on the 300 reference controls, we considered *cri,Y* < 0.04 the threshold for identifying a female fetus, while we regarded samples with *cri,Y* > 0.051 as having a male fetus. We considered samples with 0.04 <*cri,Y* < 0.051 to be gender-uncertain.

Additionally, we developed a logistic regression strategy to improve the specificity of the gender determination. We computed the probability (Pi) of that a fetus was male by the following formula:

$\text{logit}\left(p_{i}\right)=\ln\left(\frac{p_{i}}{1-p_{i}}\right)=\beta_{0}+\beta_{1}cr_{i,X}+\beta_{2}cr_{i,Y}$, where the parameters (*β0, β1, β2*) were determined by regression using the 300 reference controls mentioned above.

We regarded samples with *pi* > 0.8 as having male fetuses, samples with *pi* < 0.3 as having female fetuses, and the remaining samples as being gender-uncertain.

After gender classification, we performed XXX and XO detection on samples with a female fetus and XXY and XYY detection on samples with a male fetus.

For samples with a female fetus, we performed a t-test for chromosome abnormality detection.

$t_{i,X}=\frac{cr_{i,X}-c{r^{\prime }}_{i,X,f}}{sd_{X,f}}$ where *sd*_*X,f*_ is the standard deviation of *cr*_*i*,*X*,*f*_ − *cr*^′^_*i*,*X*,*f*_ calculated from the reference controls with female fetuses; we expected *sd*_*X,f*_ to equal zero. We considered samples with *t*_*i,X*_ or *t*_*i,X*_ < -2.5 to be XXX or XO.

For a male fetus, we first supposed that chromosome Y is monosomic and extrapolated the fitted *k*-mer coverage for chromosome X, with the fetal DNA fraction estimated only by the *k*-mer coverage of chromosome Y. We calculated the t-score by the following formula:

$t_{i}=\frac{cr_{i,X}-c{r^{\prime }}_{i,X,f}\left(1-\frac{\varepsilon_{i,Y}}{2}\right)}{sd_{X,f}}$, where *εi,Y* is the estimated cff-DNA concentration using chromosome Y data, and is the standard deviation of *cr*_*i*,*X*,*f*_ − *cr*^′^_*i*,*X*,*f*_ calculated from the reference controls carrying female fetuses with an expectation of zero. Both of these quantities are defined above.

We regarded samples with *t*_*i*_ >2.5 as being XXY or XYY. Additionally, the cff-DNA concentration estimated by chromosome X and Y independently is a combined marker for sex chromosomal aneuploidy detection especially XXY and XYY. For an XXY sample, not only was the *t*_*i*_ >2.5 but also the cff-DNA concentration estimated by chromosome X was nearly zero, with a confidence interval from −0.03 to 0.03; For an XYY samples, not only the *t*_*i*_ >2.5, but the R-value (Ratio of the cff-DNA concentration estimated by chromosome Y to that estimated by chromosome X) was nearly two, reflecting the fact that there were two copies of chromosome Y and only a single copy of chromosome X.

## Competing interest

The authors declare that they have no competing interests.

## Authors’ contributions

ZXQ, RJH, WJI and WJU conceived and designed the study; WW, ZYQ, SD, SY, ZHK, XJS, SW, YBM, ZLJ, XJH, ZZY, and LLH recruited participants and separated the plasma samples; CF, LYH, PXY, XZL, ZHY, TZM, LPP, LQY, GH, and JH performed library construction and sequencing; JFM, ZWM, CXH, LYR and LSG developed the methodology and LLF, CHF, ZCL and YYY participated in the alignment of the sequencing data and data analysis; JFM and CSP wrote the manuscript. All authors read and approved the final manuscript.

## Pre-publication history

The pre-publication history for this paper can be accessed here:

http://www.biomedcentral.com/1755-8794/5/57/prepub

## Supplementary Material

Additional file 1 **Figure S1.** The correlation between sequence GC content and relative k-mer coverage. We plotted the relative k-mer coverage of each chromosome (y-axis) among our 300 controls against the corresponding sequence GC content (x-axis). Red plot are for female fetuses and black plot are for male fetuses. Click here for file

Additional file 2 **Figure S2.** The tendency between normalized k-mer coverage and corresponding GC content. The GC content of each chromosome is listed in orders. The blue bars refer to the chromosomes that have a negative correlation between the k-mer coverage and the GC content, green bar refer chromosomes that have a positive correlation between the k-mer coverage and the GC content, and yellow bar refer to the chromosomes with no correlation. Click here for file

Additional file 3 **Figure S3.** The reconstructed relationship between chromosomes by GC content. We reconstructed the GC-content relationship between the different chromosomes by clustering the 35-mer counts for 36 GC levels (y-axis) on the different chromosomes (x-axis). The normalized 35-mer counts, as a percentage of each chromosome, are color-coded in the heat map. Click here for file

Additional file 4 **Figure S4.** The relationship between estimated cff-DNA concentration and gestational week. The black dots represent the cff-DNA concentrations (y-axis) plotted against the corresponding gestational week (x-axis) for the 443 samples with male fetuses. Click here for file

Additional file 5 **Figure S5.** The standard deviation and sample numbers. The standard deviations of the difference between the observed and fitted k-mer coverage (y-axis) for different numbers of samples (x-axis). Different chromosomes are colour-coded. The standard deviation becomes stable when the number of samples is larger than 100. Click here for file

Additional file 6 **Figure S6.** The relationship between tags number and the standard deviation of relative k-mer coverage among 150 samples. The standard deviations of the relative k-mer coverage (y-axis) declines with the increasing number of tags (x-axis) from 0.5 to 3.5 million for each chromosome. Click here for file

Additional file 7 **Figure S7.** The aneuploidy detection power estimation. The colored contour lines show the aneuploidy detection power at different gestational weeks (x-axis) and with different numbers of unique reads (y-axis).Fetal genders are shown separately. The power is much higher when the fetus is male. Click here for file
