# Supplementary figures and images for: Noninvasive Fetal Trisomy (NIFTY) test: an advanced noninvasive prenatal diagnosis methodology for fetal autosomal and sex chromosomal aneuploidies
Source: BMC Med Genomics. 2012 Dec 1;5:57. doi: 10.1186/1755-8794-5-57 (PMC3544640; doi:10.1186/1755-8794-5-57)

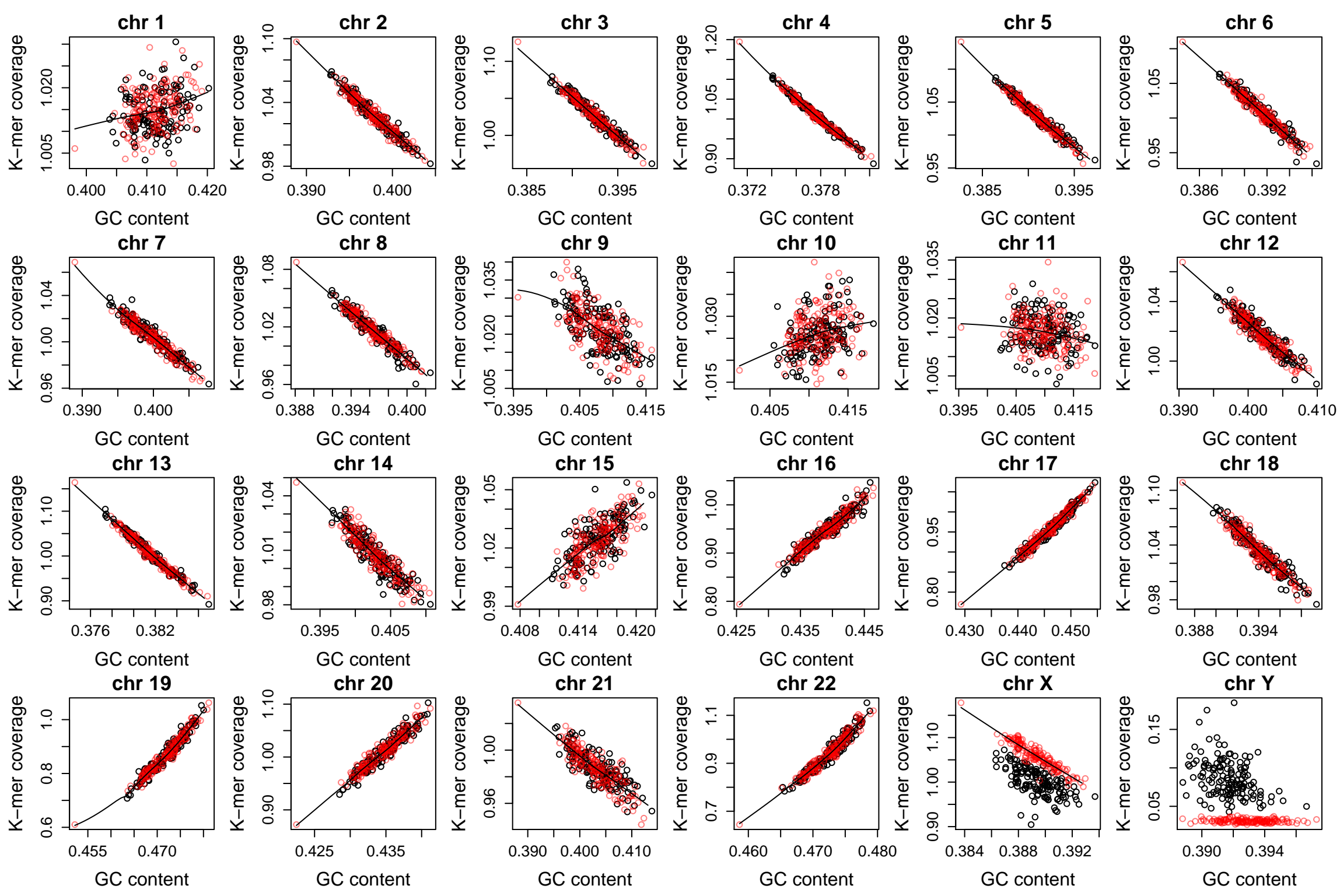

Supplement: Additional file 1 — Figure S1. The correlation between sequence GC content and relative k-mer coverage. We plotted the relative k-mer coverage of each chromosome (y-axis) among our 300 controls against the corresponding sequence GC content (x-axis). Red plot are for female fetuses and black plot are for male fetuses. [file 1755-8794-5-57-S1.pdf]

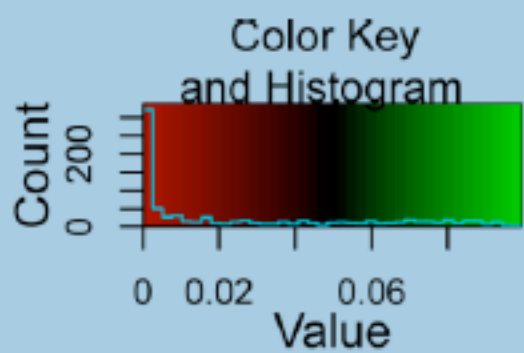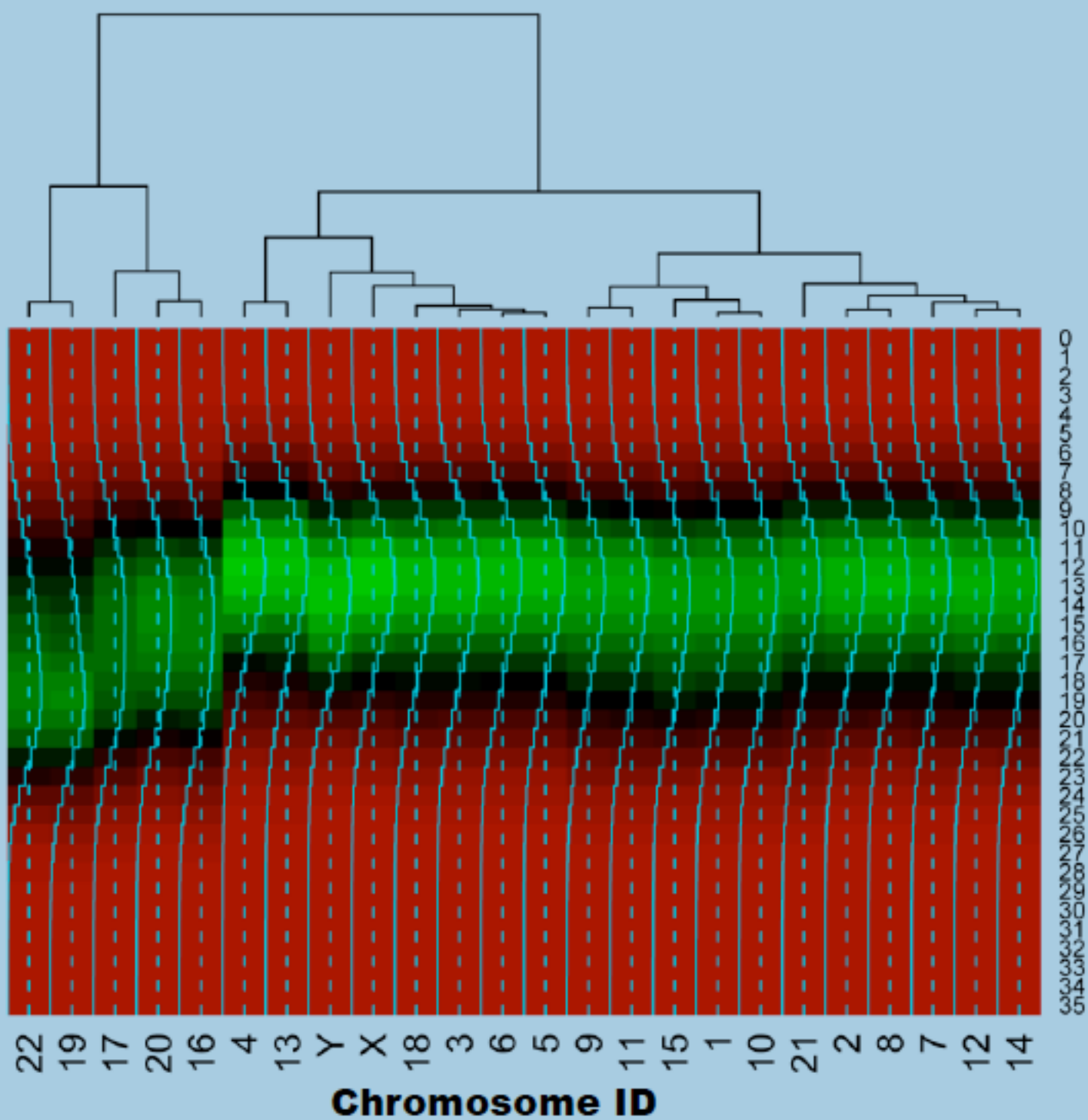

Supplement: Additional file 3 — Figure S3. The reconstructed relationship between chromosomes by GC content. We reconstructed the GC-content relationship between the different chromosomes by clustering the 35-mer counts for 36 GC levels (y-axis) on the different chromosomes (x-axis). The normalized 35-mer counts, as a percentage of each chromosome, are color-coded in the heat map. [file 1755-8794-5-57-S3.pdf]

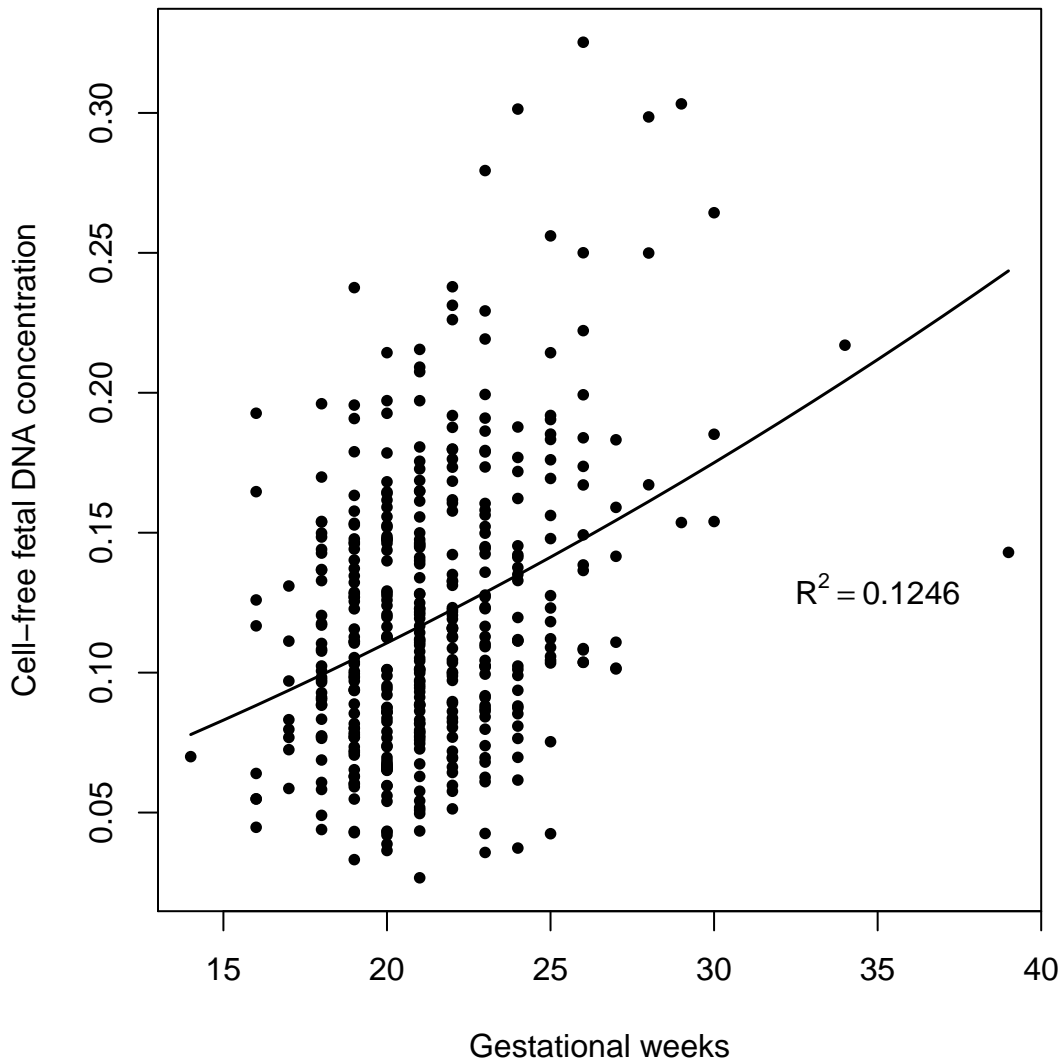

Supplement: Additional file 4 — Figure S4. The relationship between estimated cff-DNA concentration and gestational week. The black dots represent the cff-DNA concentrations (y-axis) plotted against the corresponding gestational week (x-axis) for the 443 samples with male fetuses. [file 1755-8794-5-57-S4.pdf]

Standard deviation

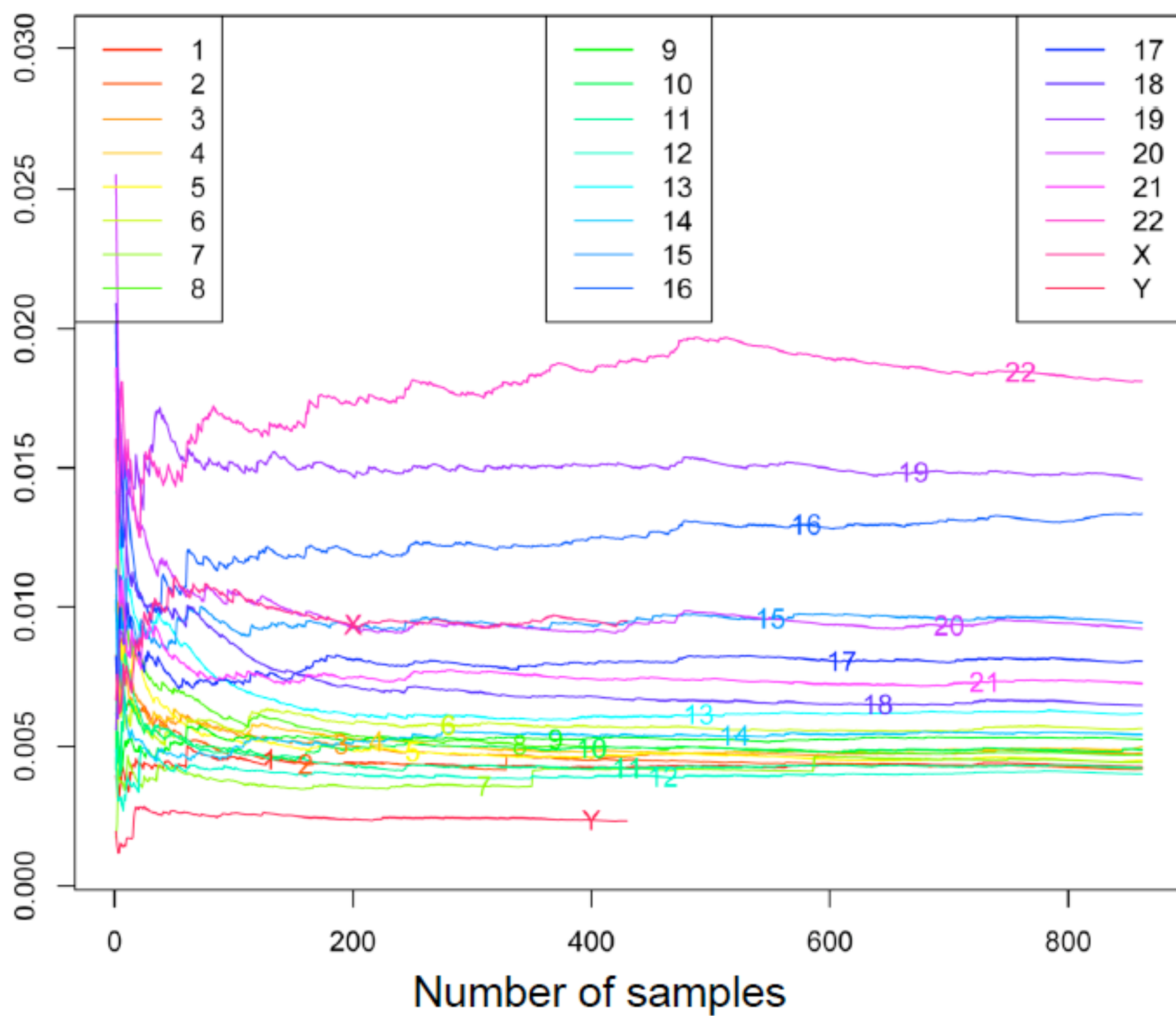

Supplement: Additional file 5 — Figure S5. The standard deviation and sample numbers. The standard deviations of the difference between the observed and fitted k-mer coverage (y-axis) for different numbers of samples (x-axis). Different chromosomes are colour-coded. The standard deviation becomes stable when the number of samples is larger than 100. [file 1755-8794-5-57-S5.pdf]
