# Supplementary material for: Noninvasive Fetal Trisomy (NIFTY) test: an advanced noninvasive prenatal diagnosis methodology for fetal autosomal and sex chromosomal aneuploidies
Source: BMC Med Genomics. 2012 Dec 1;5:57. doi: 10.1186/1755-8794-5-57 (PMC3544640; doi:10.1186/1755-8794-5-57)
Supplement: Additional file 6 — Figure S6. The relationship between tags number and the standard deviation of relative k-mer coverage among 150 samples. The standard deviations of the relative k-mer coverage (y-axis) declines with the increasing number of tags (x-axis) from 0.5 to 3.5 million for each chromosome. [file 1755-8794-5-57-S6.pdf]

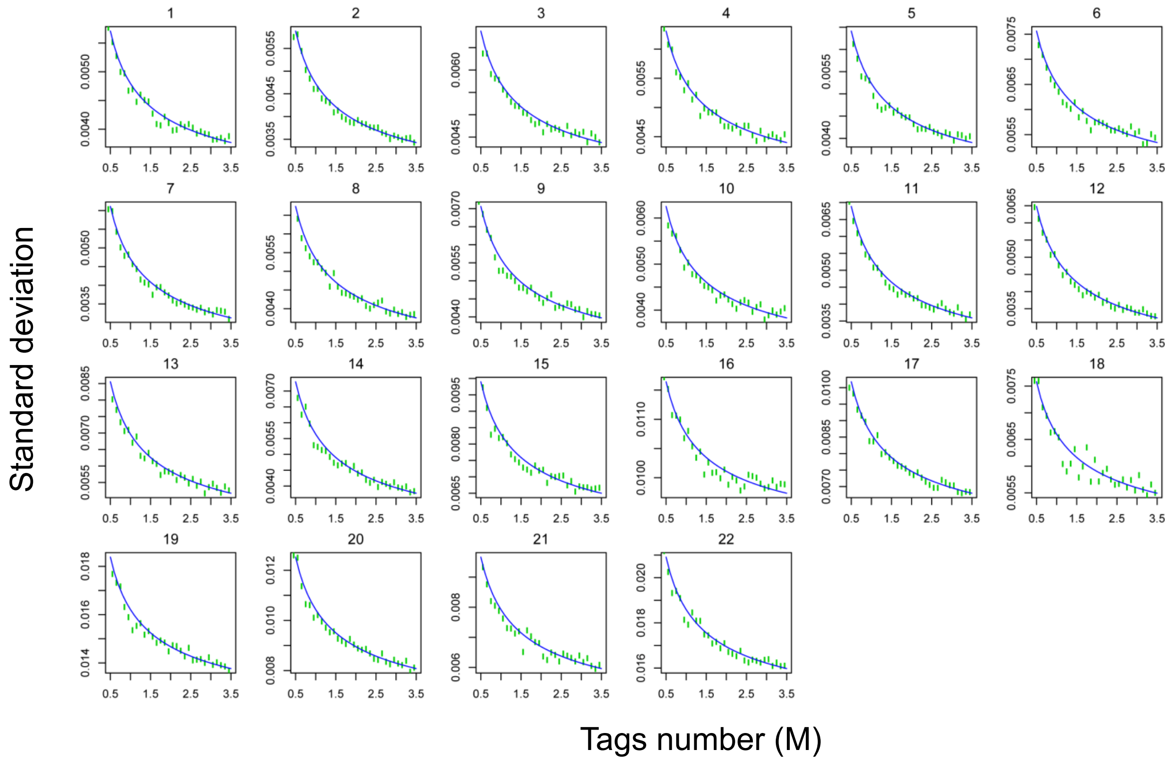

Figure S5. The relationship between tags number and standard deviation of relative k-mer coverage among 150 samples
