# Supplementary material for: Noninvasive Fetal Trisomy (NIFTY) test: an advanced noninvasive prenatal diagnosis methodology for fetal autosomal and sex chromosomal aneuploidies
Source: BMC Med Genomics. 2012 Dec 1;5:57. doi: 10.1186/1755-8794-5-57 (PMC3544640; doi:10.1186/1755-8794-5-57)
Supplement: Additional file 7 — Figure S7. The aneuploidy detection power estimation. The colored contour lines show the aneuploidy detection power at different gestational weeks (x-axis) and with different numbers of unique reads (y-axis).Fetal genders are shown separately. The power is much higher when the fetus is male. [file 1755-8794-5-57-S7.pdf]

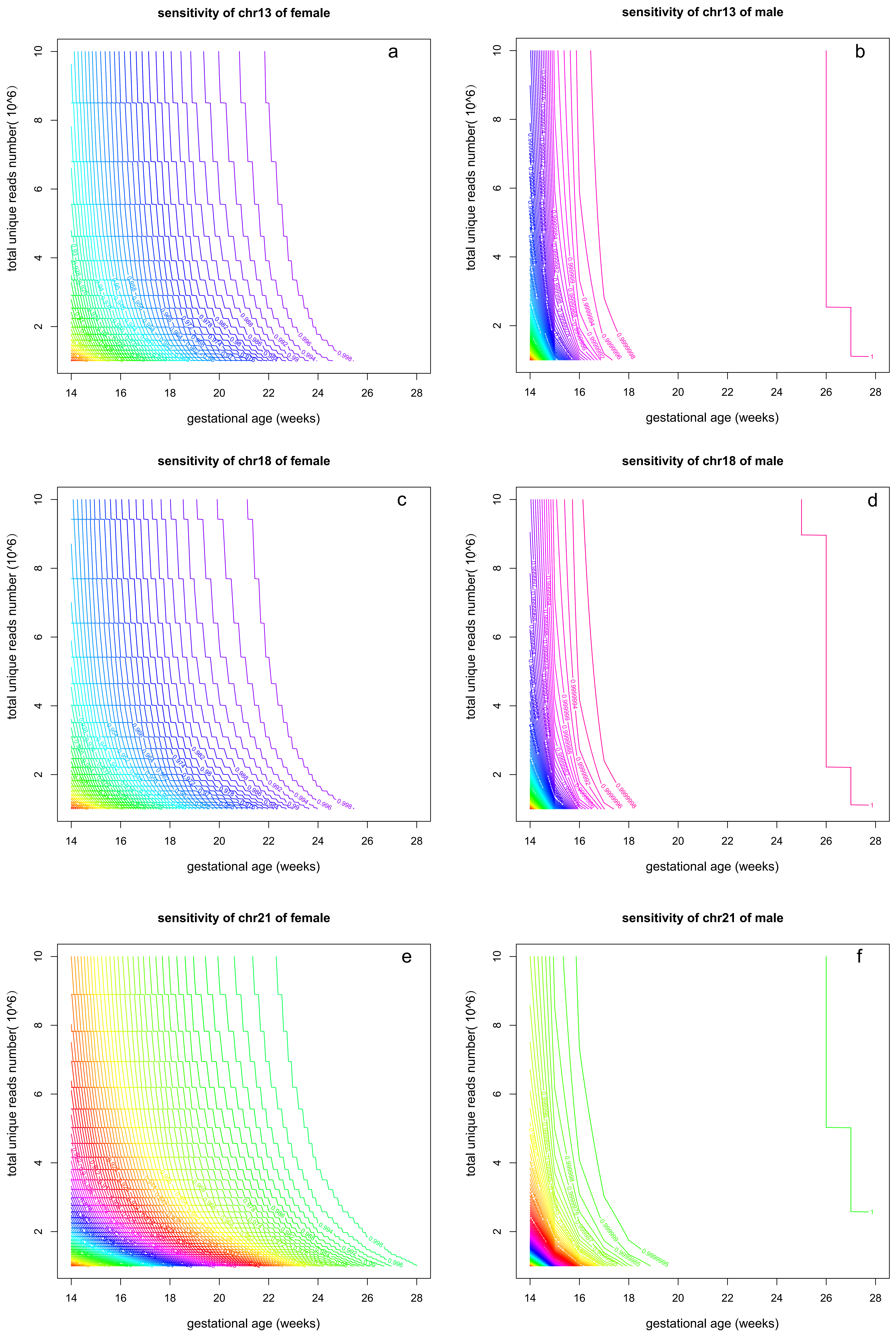

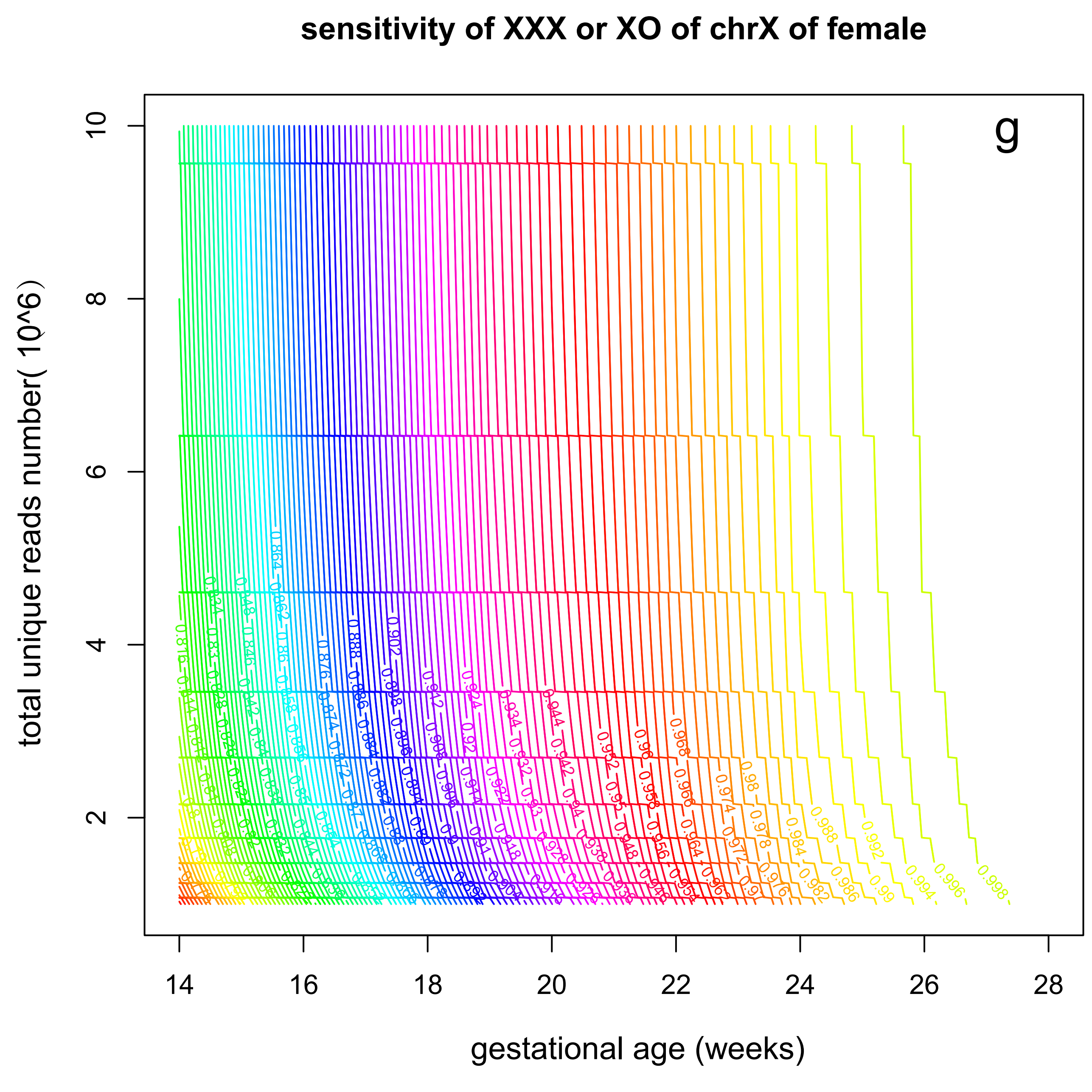

CHR \ Gender	Female	Male
T13	a	b
T18	c	d
T21	e	f
XO/XXX	g	–
